# Supplementary material for: Sparse pixel image sensor
Source: Sci Rep. 2022 Apr 5;12:5650. doi: 10.1038/s41598-022-09594-y (PMC8983698; doi:10.1038/s41598-022-09594-y)
Supplement: Supplementary file 1 — Supplementary Information. [file 41598_2022_9594_MOESM1_ESM.pdf]

Supplementary Information for

Sparse pixel image sensor

Lukas Mennel[1], Dmitry K. Polyushkin[1], Dohyun Kwak[1], and Thomas Mueller[1]*

[1]*Vienna University of Technology, Institute of Photonics, Gußhausstraße 27-29, 1040 Vienna, Austria*

*Corresponding author: thomas.mueller@tuwien.ac.at

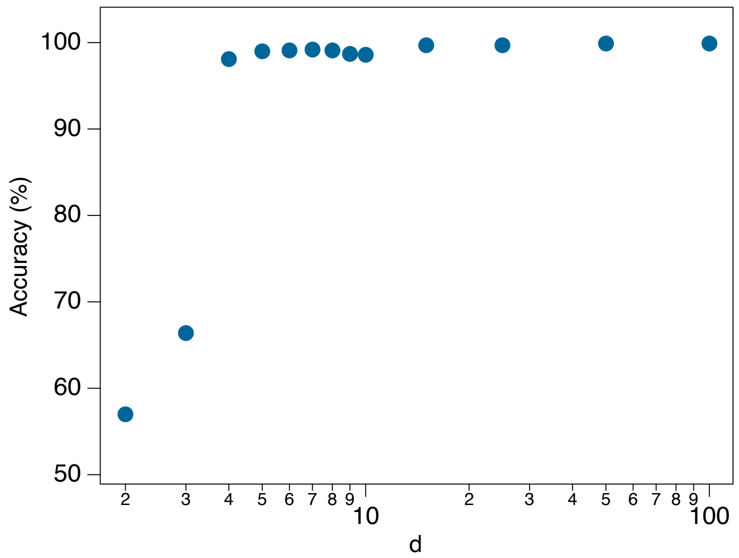

Supplementary Figure S1 | Accuracy versus number of non-zero responsivity pixels.

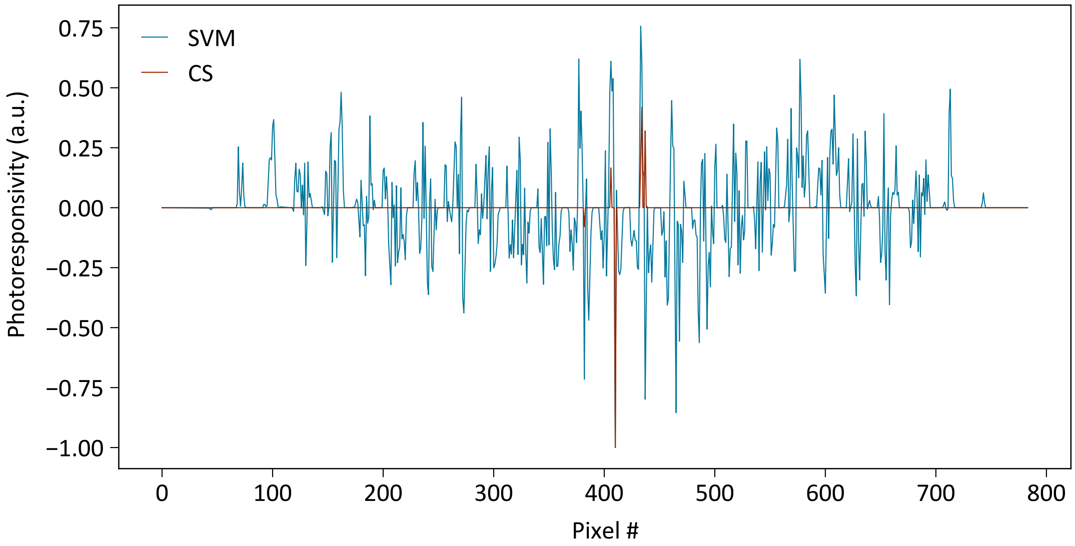

Supplementary Figure S2 | SVM and CS photoresponsivities. Data in Figures 1c and e in the main text presented as line plots.

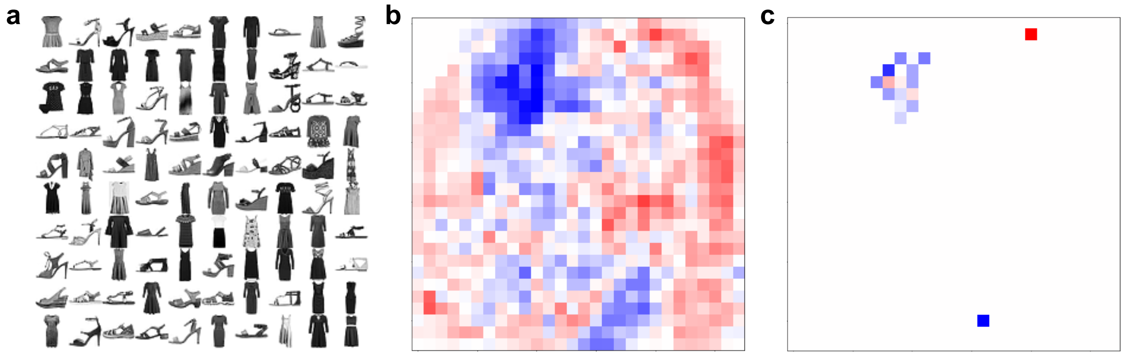

Supplementary Figure S3 | Fashion MNIST dataset. a, Fashion-MNIST is a dataset comprising grayscale images of 70,000 fashion products from 10 categories. Here, we present the results of binary classification of sandals versus dresses. **b,** Using an SVM as reference, a classification accuracy of 99.8% is obtained. **c,** A sparse pixel image sensor with only 2% active pixels achieves 98.8%.

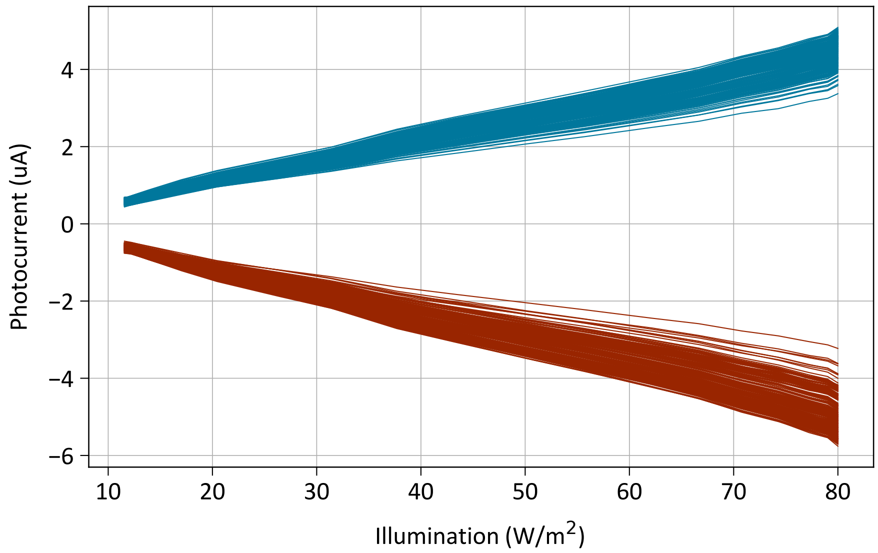

Supplementary Figure S4 | Power dependence of photocurrent.

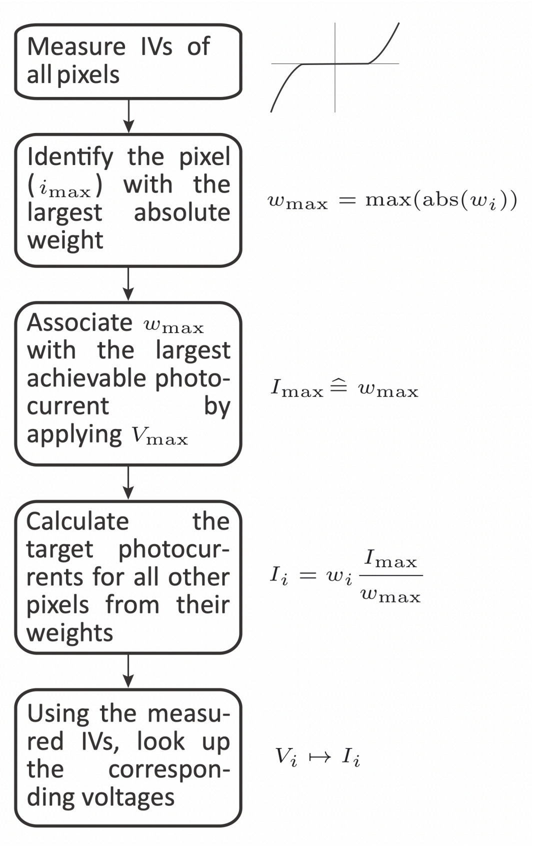

Supplementary Figure S5 | Device calibration. The flow chart illustrates how the calculated weights are implemented on the device to account for pixel-to-pixel variations.

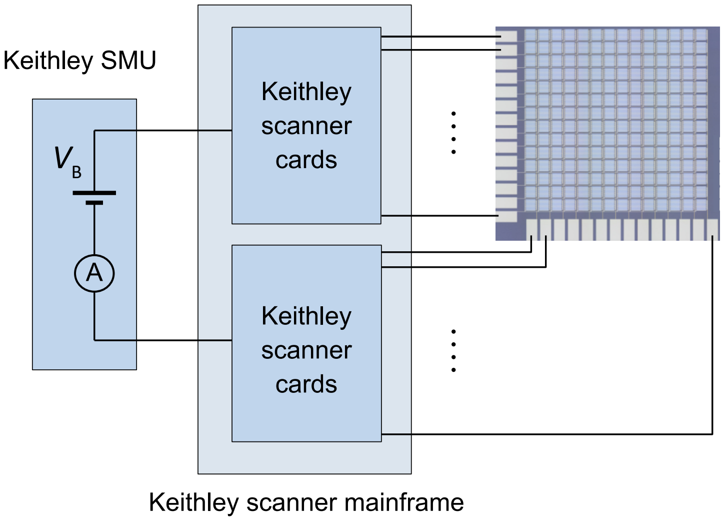

Supplementary Figure S6 | Electrical measurement setup. A bias voltage V_B, generated by a Keithley source measurement unit (SMU), is applied via a Keithley scanner mainframe equipped with low-offset current scanner cards to the respective pixel and the resulting photocurrent is integrated with the same SMU. SMU and scanner are controlled by a computer. The image sensor is mounted in a chip carrier and wire-bonded.

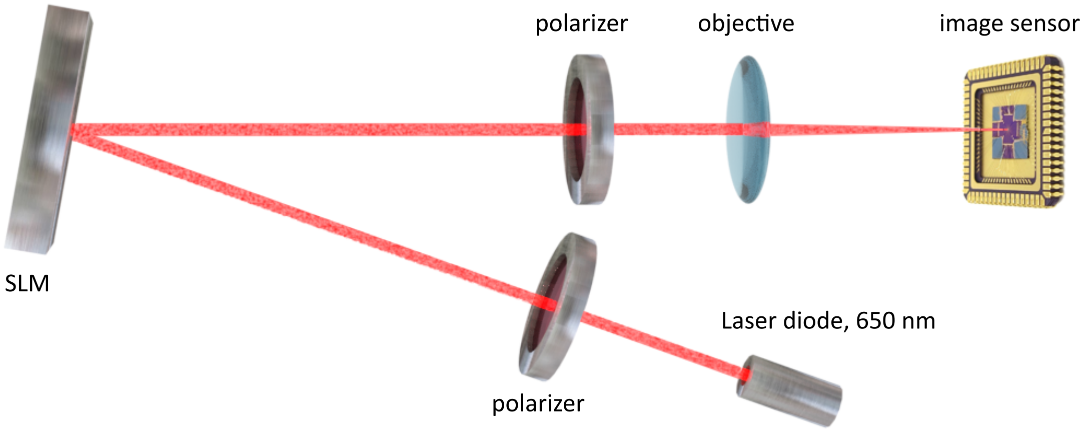

Supplementary Figure S7 | Optical setup. For description see Methods section in the main text.

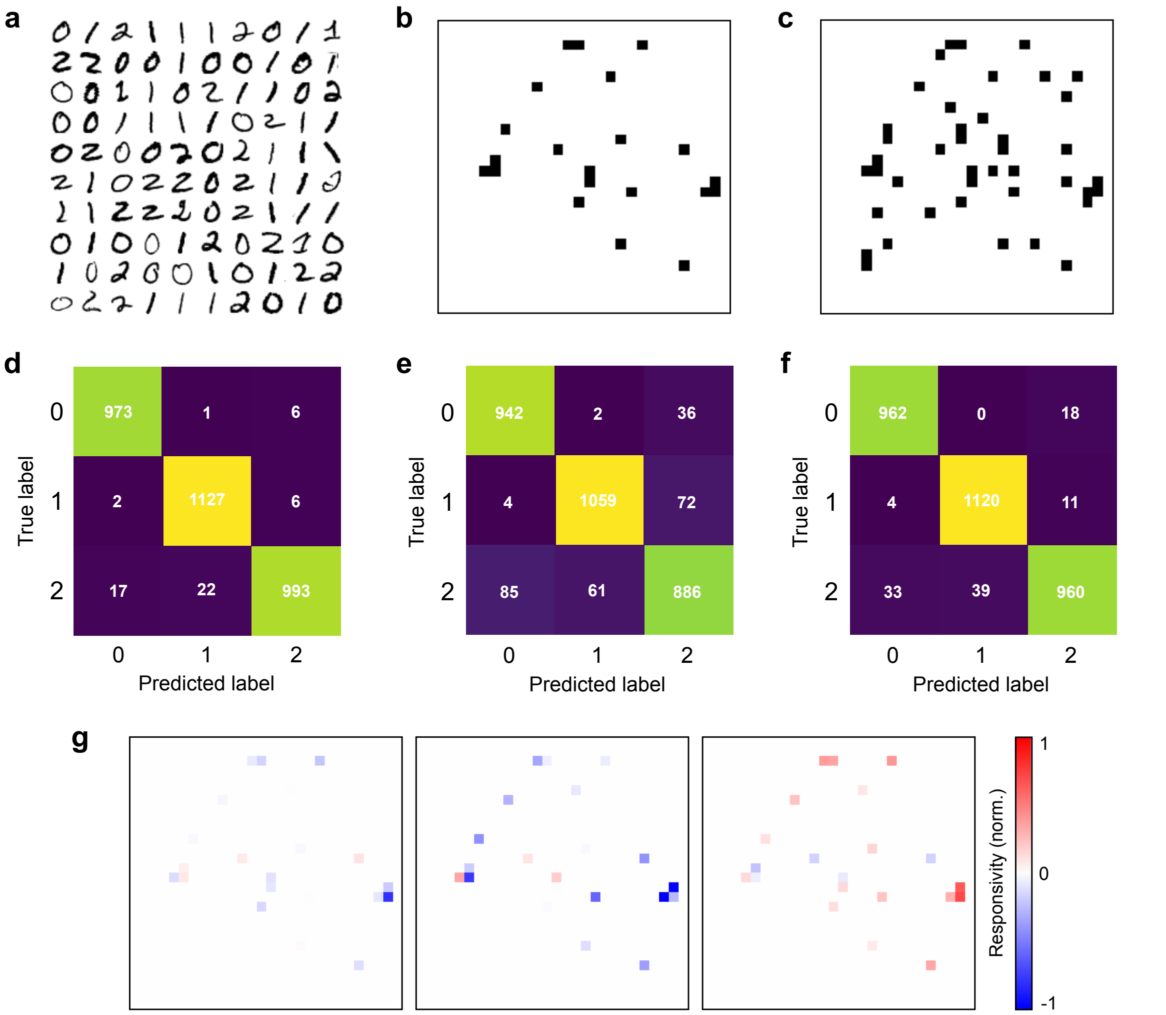

Supplementary Figure S8 | Multi-class classification. a, MNIST dataset comprising grayscale images of handwritten digits "0", "1" and "2" (3-class classification). Simulation results for CS classifiers with **b,** 2.7% active pixels and **c,** 5% active pixels achieve classification accuracies of 92% and 97%, respectively. The corresponding confusion matrices are shown in **e** and **f**, respectively.

Using a SVM as reference, a classification accuracy of 98% is obtained. The SVM confusion matrix is presented in **d**. **g,** Each pixel is divided into $c = 3$ subpixels with shown responsivity values. As in the binary case, one pixel after the other is addressed, and three voltages are applied to set the responsivity values of each of the subpixels simultaneously. The three output currents are integrated and the class corresponding to the largest total output is chosen. The responsivities are calculated[7] according to $\min_{\mathbf{r}} ||\mathbf{r}||_1 + \lambda||\mathbf{rc}||_1$ s.t. $||\mathbf{\Psi}^T\mathbf{r} - \mathbf{w}||_F \leq \varepsilon$, where $\mathbf{c}$ is a column vector with $c - 1$ ones, ε is the error tolerance, λ is the learning rate, and $||\cdot||_F$ denotes the Frobenius norm.

```
import matplotlib.pyplot as plt
import numpy as np
from tensorflow.keras.datasets import mnist
from pysensors.classification import SSPOC
from sklearn.linear_model import SGDClassifier
from sklearn import metrics

# threshold parameter; set between 0 and 1; determines # of pixels
threshold = 0.65

# set seed
random_state = 0

# get MNIST
(X_train, y_train), (X_test, y_test) = mnist.load_data()

# 2-class classification
digits = [0,1] # select two digits
train_filter = np.where((y_train == digits[0] ) | (y_train ==
digits[1]))
test_filter = np.where((y_test == digits[0]) | (y_test ==
digits[1]))
X_train, y_train = X_train[train_filter], y_train[train_filter]
X_test, y_test = X_test[test_filter], y_test[test_filter]

# reshape
train_samples = y_train.shape[0]
test_samples = y_test.shape[0]
X_train = X_train.reshape(train_samples,784)
X_test = X_test.reshape(test_samples,784)

# classifier (SVM)
classifier = SGDClassifier(max_iter=5000, loss='modified_huber',
random_state=random_state)

# fit model
model = SSPOC(classifier=classifier)
model.fit(X_train, y_train, quiet=True)

# select pixels
model.update_sensors(threshold=threshold*np.max(np.abs(model.sensor_
coef_)), xy=(X_train, y_train))

# inference on test set
y_pred = model.predict(X_test[:, model.selected_sensors])
print('Accuracy:', metrics.accuracy_score(y_test, y_pred))

# plot relevant pixels and their weights
image = np.zeros(784)
image[model.selected_sensors] = classifier.coef_
plt.figure(figsize=(10,10))
plt.imshow(image.reshape(28, 28))
plt.colorbar()
```

Supplementary Information S9 | Python script for binary classification. In the multi-class case, regularization can be implemented through: `model = SSPOC(threshold=threshold, l1_penalty=l1_penalty, classifier=classifier)`
